# Editorial and Miscellaneous

**Published:** 1863-09

**Authors:** 


### EDITORIAL AND MISCELLANEOUS.

### WORK FOR THE MEDICAL SOCIETIES.

It is capable of demonstration that something more can be done by the Medical Societies than to expel disorderlies and administer condign punishment to recusants. We question whether even the expulsion of Surgeon-General Hammond, as demanded by our Ohio friends, would fully accomplish the "mission" of the Medical profession.

It is doubted by the present writer whether any number of expulsions, suspensions or admonitions would, alone, bring the dignity of the profession up to the highest attainable point. The "casting out of devils" is scarcely all there is, even of the Christian religion—we do not know that that alone exhausts the offices of Medical Association.

Medicine in these later years, especially since Schleiden and Schwann pointed out the wonderful functions of the organic cell, has been disposed to become mainly microscopic in its observations. It is time that it began to "triangulate the great spaces." The civil war with its gigantic arrangements tends to enlarge the views of men—physicians and all. Mere cutting off of arms and legs, extraction of bullets, ligation of arteries, and sewing up of wounds, has been proved but a very infinitesimal part of the duties even of the Army Surgeon. The physician still ranks the surgeon practically although not in popular éclat.

Fortunately at the present time we have at the head of the Medical Staff of the Army a man who is not only minute and accurate in observation, but also of large mental grasp and comprehensive view.

As a proof of this we only need to point to the vast hospital arrangements he has perfected—to the history of the diseases and wounds of the war he has inaugurated, and the magnificent collection of material for future generalization and discovery which he has commenced in the National Army Museum.

In our view the great want of the time is that physicians in civil practice, "wheresoever dispersed," should systematize their efforts towards the same end in civil observation which is now attempted in the military branch of the profession.

It were scarce exaggeration to say that the number of lives saved and amount of general health secured, as the result of the hygienic knowledge gained and sanitary principles impressed by the records of the war, will eventually more than counterbalance the lives lost and health destroyed by this same terrific struggle.

"There is a soul of goodness even in things evil." Systematic, rightly organized effort will accomplish almost miracles. Individuals can do but little—masses of determined men can do almost everything. "Extraordinary cases" which make up so large a proportion of the communications to our Medical journals are comparatively valueless. A solid structure of Medical science cannot be constructed from such cobwebs.

Medical men, in common with all scientific investigators at the present time, recognize the truth that inquiries with reference to physical laws, in order to give results of real magnitude, must take a wider and more comprehensive range than can be attempted by any single, although it be a master, mind. As the great laws which govern the apparently eccentric phenomena of nature, such as the winds, the hurricanes, the fall of rains, earthquakes, and all other meteorological and terrestrial changes, can only be determined by widely extended observations and multitudinous observers, girdling and investing the entire planet, thus do we believe that disturbances of Plato's Microcosm, which occur apparently so disorderly, as to justify the old mythologies in attributing them to the wrath of offended deities, are nevertheless under the orderly control of laws, discoverable by general and continuous observations over all the continents, islands and seas.

All the appliances of art and science should be brought into requisition, and as the students of meteorology follow up the track of the winds, and vapors, and storms, laden with thermometers, barometers, hygrometers, *et id genus omne*—so should the Medical profession follow up the trail of disease. Systematic, comprehensive action is what is needed. If the Medical profession throughout the United States were organized as are their brethren in the Army and Navy, and all cases treated, whether resulting in recovery or death, duly reported to some central bureau, there to be subjected to analysis and scientific examination, under all the light which modern science is capable of throwing upon them, there can be no doubt that in a very brief period Medicine as a science would take strides in advance unexampled in all its thousands of years' history.

What a light would shine all along the track of epidemics —endemics would disclose their strange secrets, and sporadic diseases would no longer surprise like arrows falling in the darkness.

Under our particular form of government it is, perhaps, impossible to compel the general collection of these details from which the body of an exact and generous science could be built up. Petty individual jealousies and the poisonous rancor of political parties would prevent in any event. But in this age of the world what there is a necessity should be done, will find a way to have itself done.

The work must be done by individuals, the results gathered up by associations, legal or voluntary, or both. This is the true work of Medical Societies, town, county, state, national, continental or cosmopolitan. When they address themselves to this work, we pledge ourselves to join them all.

Just now we have little respect for the Trades-Unions, and punitive leagues, which too often take the name of medical societies—(*lucus a non lucendo*).

We shall return to this subject in an ensuing No. and point out what we believe to be feasible and practicable methods of operation. The time has come when facts and not merely the attenuated figments of fancy, or the mouldy and effete dogmas of past ages, are to be the elements of thought.

---

*Return of Prof. Miller from Europe.*—By a letter just received, we are informed that Prof. Miller, after a peculiarly pleasant and professionally profitable tour, has engaged his return passage by the Persia which leaves Liverpool on the 26th inst. He will, therefore, be present at the opening lec tures of the College.

---

*Rush Medical College.*—With the last number of the *Journal* we sent to each of our subscribers the Twenty-first Annual Announcement of Rush Medical College. We take occasion here to say that the prospects of the College were never more flattering than at the present time. The Secretary informs us that the number of letters received, thus far, surpasses very largely those of any former year.

To our own knowledge, each member of the Faculty has made the most strenuous efforts to increase the facilities for communicating instruction, and to illustrate his teaching.

No resort has been, or will be, had to personal solicitation of students, underbidding, or other unworthy acts, *sometimes*, we regret to say, made use of by competing institutions. Rush

Medical College relies solely upon the character of its teaching, as demonstrated by the success of its graduates in both civil and military life.

The ambition of its Faculty is to keep in the front rank of *true* Medical progress, recognizing this as the highest medical conservatism. The determination is that the lectures given shall not be a mere rehash of the text books, but thoroughly brought up to the times, with an unmistakable vitality of their own.

---

*The Pharmacopœia of the United States of America. Fourth Decennial Revision. By Authority of the National Convention for Revising the Pharmacopœia, held at Washington, A. D.*, 1860. *Philadelphia, J. B. Lippincott & Co.* 1863.

The usual decennial Convention for revising the Pharmacopœia met at Washington on Wednesday, May 2d, 1860, and a full delegation responded to their names. George B. Wood, M. D., of Pennsylvania, (*clarum et venerabile nomen*,) was appointed President. Jacob Bigelow, M. D., of Mass., and Edward Warren, M. D., of S. C., respectively first and second Vice Presidents; Thos. Miller, M. D., of D. C., Secretary, and Jno. C. Riley, M. D., Ass't Secretary.

As the result of the action of this Convention, numbering many of the most distinguished members of the profession in the Union, we have the book before us.

The Convention was governed by the same rules adopted by the Convention of 1850, and provided for a General Convention on the first Wednesday of May, 1870.

The revised Pharmacopæia is published in cheap form so as to ensure its general use by physicians and apothecaries.

We notice in the index that the quantity of the syllables is so marked that it will serve as a pronouncing vocabulary of the Materia Medica.

The changes found necessary in the Pharmacopœia from the progress of the Science, were quite numerous. Some additions have been made, many alterations in arrangement and details, and some articles have been displaced from the list.

Especial attention has been paid to mechanical methods, e. g. Percolation, Fineness of Powders, Weights and Measures, Solutions, &c. Several changes in nomenclature, involving both the Latin and English names, are to be noticed, and, where possible, we observe the singular number has been preferred to the plural. From such casual notice as we have been able to give, we believe that the Convention have well discharged the onerous duty imposed upon them.

---

*The Reform School.*—Under its latest *alias*, the Reform School has secured the kindly offices of one Follansbee, an ice peddler, who has cheerfully assumed the cast-off ornaments of Mr. Lind, late Commissioner of Sewerage, and turns up as a patron of the profession—provided they pay him for a part of his kitchen garden, and interest on a little building which he had prudently erected to help pay taxes. In default of payment of interest, it is understood that the building will revert to its original design, a saloon and day boarding house for cattle drovers and experts in the hog market. Its immediate vicinity to the immense railroad cattle yards, peculiarly adapts it to this purpose. Whilst occupied as a Medical College, its propinquity to that odoriferous part of our city suburbs, it is believed, will render it peculiarly convenient for the illustrative necessities of the Prof. of Comparative Anatomy, and the Prof. of Public (and Medical College) Hygiene. Meanwhile the family of the iceman will have full opportunity to inspect the dissecting room from their back windows, and " nose it behind the arras " of the rear parlor. Another advantage is that it joins walls with the district police station, which will facilitate the maintenance of order among the multitudes who will throng its spacious halls, and besides this the clubs of the police, if exercised as heretofore in that neighborhood, according to the records of our Courts, will go far to supply any hiatus in the *materiel* department.

*Fibrin us Coagula in the Heart.*—There is a great deal of scattered experience with regard to this interesting subject which should be collected. Dr. J. F. Miner, editor of the *Buffalo Journal*, in an interesting article entitled, "Intra-cardiac Blood Concretion," adduces two cases going to show the presence of these formations in death from chloroform. He makes these pertinent suggestions:

"It is perhaps an entirely new view of the dangers of chloroform to regard it as liable to produce death by inducing blood-clot during the periods of suspended respiration which so often occur under its influence; still, in connection with hæmorrhage, which is supposed also to favor this formation, it may have more influence than generally supposed. Whatever interrupts the circulation, or greatly enfeebles it, must certainly be regarded as more or less operative in producing such result."

In which connection we beg leave to call Dr. Miner's attention to our own remarks, under this head, on page 332 (July No.) of this volume. Any notable reduction of the heart's action directly favors formation of these coagula, and their subsequent danger. The therapeutic and prophylactic indica-ions are obvious. Not only death but "complications," in the way of local disorders, are exceedingly liable to occur from the formation and detachment of these coagula into the current of the circulation. We repeat: "He who boasts that with Veratrum Viride, or Tartar Emetic, or other sedative, he has reduced the pulsation from a hundred, or more, below the normal standard, by no means convinces me that, unwittingly perhaps, he is not responsible for the sudden, or it may be slow and lingering, death which ensues." The attempt to control pulsation should, mainly, if not wholly, be through endeavor to remove the cause whatever that may be. The heart labors more rapidly in consequence of the demand made upon it, just as its muscularity increases when there is permanent obstruction. The hypertrophy is not disease—the more rapid stroke is not disease! This thing is worth looking after.

*Tetanus.*—A boy of seventeen placed a common Chinese cracker in a blow-gun and, having lighted the string, placed one end of the tube in his mouth so as to blow it at some object. Unfortunately the cracker exploded prematurely, and passing into the mouth perforated the body of the tongue, making a hole half an inch in diameter. The wound bled at first profusely, but this speedily subsided and was followed by severe inflammation. On the third day a piece of the cracker, which had remained undiscovered in the tongue, was discharged, and on the sixth day the tongue seemed rapidly healing. On the eighth day spasmodic movements were noticed about the muscles of the jaw, but these subsided mainly, after the operation of a cathartic and the use of anodynes. Careful examination disclosed no particular cause of irritation in the part. But on the tenth day general convulsions and persistent lock-jaw supervened. Chloroform readily controlled the convulsions, but on its suspension they speedily returned. He was kept under its influence fifteen hours, and then succumbed.

---

*Army Diarrhœa.*—Surgeon GEO. WINCH, M. D., of the 29th Wisconsin Vol., commends in the diarrhœa now prevailing at Vicksburg: ℞. Pulv. Opii, Quiniæ Sulph., P. Gum Camph. Capsicii, *aa* gr. j; Sodæ BiCarb. gr. viij. M.

---

*Lupus.*—JOHN HASTINGS, M. D., of the U. S. Marine Hospital at San Francisco, reports in the *Pacific M. & S. Journal* three cases of Lupus, strongly marked, cured by the assiduous local application of Stramonium. He applied a poultice of the bruised fresh leaves, and also the ointment prepared from the same. One case was cured in five, another in six, and the other in eight weeks. The diagnosis was confirmed by other medical gentlemen, and his description is clear and exact.—There was nothing peculiar in the internal treatment. The patients were males, respectively 30, 35 and 41 years of age. Dr. B. thinks this method likely to prove successful in many cases otherwise deemed incurable.

*Bitters.*—A correspondent wishes to ascertain the composition of a popular tonic and stomachic known as Swain's Bourbon Bitters. A note from the manufacturer informs us:

"The composition of Swain's Bourbon Bitters is as follows: Orange Peel, Camomile Flowers, Cardamom, Calamus, Ginseng, four ounces of each; Bourbon Whiskey, free from Fusel Oil, 40 Gallons."

This is a vast improvement on the heterogeneous mixtures in vile, half-distilled whiskey, or worse rum, generally palmed upon the bibulous or invalid portion of the public. The manufacturer exhibits a praiseworthy confidence in the character of the menstruum he employs by giving publicity to the formula. Most of the "bitters" in the market are but adroit combinations to disguise the physical properties of the worst possible alcoholic tinctures of Fusel Oil. Every physician should warn his patients against their use.

---

*Variola.*—The small pox has lately made terrible havoc on the Pacific coast, both north and south of San Francisco. The Victoria papers mention that its ravages have been excessive among the Indians of Vancouver's Island (over 1,000 died.) In the southern part of our State, at Los Angeles for instance, the epidemic has also prevailed extensively.—*Pacific Journal.*

---

*Lotion for Indolent Ulcers.*—The following lotion will be found serviceable in indolent ulcers of the legs, etc.: ℞. Calcis chloridi ʒ i; Pulv. Opii ʒ iss; Aquæ ℥ vi. Fiat lotio et cola. Sig. Keep the ulcers wet with it by means of lint.—*Id.*

---

*Osteocopic Pains.*—*Periostitis*, *Nodes.*—We have been in the habit of using for many years the abortive method advocated for buboes by Dr. Malapert, a French army surgeon, in cases of syphilitic osteocopic pains, periostitis and nodes affecting the tibia, ulna, clavicle, sternum and cranium.

A blister the size of a silver quarter of a dollar is applied over the painful bone, and a pledget of lint soaked in a solution of corrosive sublimate (gr. xv to xx for ℥ i of water) is placed over the previously denuded vesicated surface and allowed to remain in situ for an hour. The parts are afterwards dressed with simple cerate. Considerable pain is caused by the caustic application, but the relief which follows is much more permanent than that attained by any other method we are acquainted with. The patients generally request of themselves a second or third application when necessary. In connection with the internal administration of iodide of potassium, we know of no superior treatment in these affections. In some obstinate cases, however, mercurial vapor baths twice a week may be added.—*Id.*

---

*Liquors and Wines for Medicinal Use.*—In these days when everything which can be adulterated, imitated and sophisticated is thus palmed off upon buyers, it becomes the purchaser to closely scrutinize the source of everything of a manufactured kind which is offered for sale. This remark is especially true with regard to the various preparations of alcohol. Unfortunately the price is no criterion of the quality, and few practitioners, or even druggists, have the conveniences, or the manual skill, requisite for satisfactory analysis. The consumer is at the mercy of the dealer.

Without any intention of writing an essay upon this hackneyed subject, we merely call attention to the point that without invidious comparisons Messrs. Hoyt, Pierce & Co., No. 147 S. Water st., in this city, are gentlemen whose representations with regard to their wines and spirits may be fully relied upon. They supply from their immense stock some 300 of the first class druggists of the North West. Our correspondents, who have from time to time written us for a reference in this matter, may take notice and govern themselves accordingly.

---

*Noisy and Restless Insanity.*—*Hydrocyanic Acid.*—A case is related in which the patient was noisy and restless, incessantly in motion, constantly shouting and screaming, and for long periods sleepless. She was completely delirious, and had not during the day a moment's repose. Four minims of dilute hydrocyanic acid were given every hour, in half an ounce of camphor mixture. Immediately after the first dose she became quiet and still, and slept at night. She continued better until the medicine was discontinued, when she at once relapsed to her old state. This may prove a valuable hint in some of these distressing and unmanageable cases.—*Dr. K. McLeod.*

*Quinine.—Therapeutical Action.*—Quinine is held to be a direct tonic on insufficient grounds, its real action is that of a sedative to the efferent nerves of the sympathetic, for the following reasons: That it requires a very much larger dose of quinine to produce its physiological effects in robust persons than in the debilitated; and in extreme asthenia, even small doses are poisonous. That it decreases the force and frequency of the pulse. When given in inflammatory fever, it restores the activity of the secretions. The following practical points will be found true. Quinine is only useful in dyspepsia, which results from suppression of the digestive secretions from febrile irritation. It should be used rather in sthenic than asthenic cases of inflammation. It is not suitable in diseases of the heart.—(Mr. R. Walker,) *Med. Times and Gazette, Feb.* 21,'63.

### TO THE MEDICAL PROFESSION OF ILLINOIS.

The undersigned was at the late meeting of the Ill. State Medical Society appointed as Chairman of the committee on Drugs and Medicines.

All members of the Medical profession in the State of Illinois are hereby requested to report the result of their observations in regard to any new remedies, or new application of those long known, to the Chairman at Quincy, Dr. R. G. Laughlin of Hayworth, or Dr. F. R. Payne of Marshall.

Resp'y, F. K. BAILEY,
Ch'm'n Com. on Drugs and Medicines.

General Hospital, Quincy, Ill., Aug., 1863.

☞ Business letters to the Journal should be addressed to Dr. E. INGALS, Chicago, Ills., P. O. Drawer 5787. When money is received a receipt will be returned with the next number of the Journal, and subscribers failing to get such receipt will confer a favor by giving us notice of the omission.

Vaccine Matter.—Fresh and reliable Vaccine Matter may be obtained by enclosing One Dollar and addressing
Chicago Medical Journal,
P. O. Drawer 5787, Chicago, Ill.

